# Supplementary material for: Determinants of personal vaccination hesitancy before and after the mid-2021 COVID-19 outbreak in Taiwan
Source: PLoS One. 2022 Jul 29;17(7):e0270349. doi: 10.1371/journal.pone.0270349 (PMC9337646; doi:10.1371/journal.pone.0270349)
Supplement: Attachment — Submitted filename: response.docx [file pone.0270349.s001.docx]

Authors’ response

We deeply thank the referees for their insightful and helpful comments on our paper. We believe they have helped us to improve the paper. We have made according revisions to the manuscript.

In order to meet the requirement of the reviewer, a significant change to the paper is that we rename the title to “Determinants of personal vaccination hesitancy before and after the mid-2021 COVID-19 outbreak in Taiwan.” We believe this title is more consistent with the content of the manuscript too.

Moreover, in order to meet the requirements from the journal, we made some cosmetic changes of ethic statements in the new manuscript too.

Below we respond to individual comments from the referees. We use black text for the reviewers’ comments and our reply in blue. In the manuscript, we use blue color for the text we added.

Reviewer #1:

Thanks a lot for the opportunity to review this interesting and timely manuscript. In this study, Hsuan-Wei Lee et al, investigated COVID-19 vaccine hesitancy in Taiwan and its associated factors. This is a timely study considering the recent record number of COVID-19 cases in Taiwan.

The study design is robust, and the authors used a cross-sectional national survey before and during the first noticeable increase in the number of COVID-19 cases in Taiwan between March 2021 and May 2021 to assess the attitude of the general population to COVID-19 vaccination with a large sample size. Overall, the manuscript is well-written, and the findings represent a significant contribution to the field of COVID-19 vaccine hesitancy. The manuscript is well organized and is comprehensively described and it was written in correct and readable language. The research design was proper, the methods were adequately described, the results were presented clearly with sufficient Tables, and the conclusions were supported by the results. Importantly, the potential limitations were presented clearly by the authors.

Minor comments:
1. The authors can benefit from revising the title which can be a bit vague for the readers from countries where the first wave (outbreak) of COVID-19 occurred in the first half of 2020 rather than 2021.

We agree with the reviewer that the old title is a little bit unclear. Following the reviewer’s suggestion, we change the title of this article as “Determinants of personal vaccination hesitancy before and after the mid-2021 COVID-19 outbreak in Taiwan.” We deeply appreciate the reviewer’s valuable suggestion.

2. In the Introduction section, lines 9-18, the authors can benefit from two additional reference on COVID-19 vaccine acceptance rates worldwide and in Taiwan:
A. <https://doi.org/10.2147/JMDH.S347669>
B. <https://doi.org/10.3390/ijerph18115579>

We thank the reviewer’s suggestion. The two additional references are indeed relevant to this research and they are both added in the new manuscript (line 14). We also added two supplementary references to illustrate the initial success of controlling the COVID-19 epidemic (line 27).

3. In the Introduction section, lines 33-37, the authors can benefit from adding more information regarding the number of cases and mortalities that occurred during the first wave (if it can be called so) of COVID-19 in Taiwan in May 2021.

We appreciate the reviewer’s suggestion. The information regarding the number of cases and mortalities are now provided (line 36-40).

4. In the Introduction section, line 39: please add a reference to support this statement.

We thank the reviewer’s suggestion and add a reference to support the statement in the new manuscript (line 45).

5. In Figure 1, please add the reference from which this relevant data was retrieved.

We thank the reviewer’s suggestion. The dataset we referred to is cited in the new manuscript (line 64).

6. In Table 1, please add a footnote to spell out the abbreviations (e.g. WHO). The same applies for Table 2 (USD)

We appreciate the reviewer’s reminder and spell out the two abbreviations we used in the new manuscript.

7. In the Discussion section, the authors can benefit from adding a paragraph to compare the acceptance rate to COVID-19 vaccination in Taiwan to that in neighbouring countries which were much higher (e.g. China 82%, South Korea 77%) using the aforementioned reference: <https://doi.org/10.2147/JMDH.S347669>

We thank the reviewer’s suggestion and think it is indeed important for us to make a comparison of vaccination acceptance rates to the nearby countries. However, the reason Taiwan has a relatively low acceptance rate to COVID-19 vaccination in this study is mainly because that the survey was done in the period that there was no local COVID-19 cases spreading in Taiwan for several months. When the pandemic is eminent, people’s vaccination acceptance rates would grow much higher, and this is also consistent to our main finding in the manuscript. Our results showed that participants’ vaccination intent was highly sensitive to time and the risk of infection people perceive. To put elaborate on this more clearly, we added several sentences in the new manuscript accordingly (line 28-30 and 259-262).

Reviewer #2: Comments to the Author
My comments are as follows:

1. Abstract- Well structured and summarizes the overall purpose of the study and the research problem(s) investigated. The basic design of the study; major findings and trends found as a result of the study is also showcased.

2. Materials and methods- The authors have included a proper survey questionnaire along with the assessment scale to analyze their objectives.

3. Statistics: The involvement of regression methods (logistic and linear models) provides clearance of bias that may have generated by the different variables. The confounding/impending factors are well neutralized by the statistical methods.

4. Discussion: The discussion requires more additional statements with regards to the existing literature search. Include the following references and citations reflecting the COVID-19 updates to strengthen the manuscript:
- Origin and transmission (use reference and cite:  doi:10.1136/postgradmedj-2020-138234)
- To mention in brief about vaccines (use reference and cite: “doi: 10.1136/postgradmedj-2021-141365. AND  doi:10.1136/postgradmedj-2021-140654”)

- Definition of vaccine resistance and hesitance (use reference and cite: doi:10.3390/vaccines9101064).

- Compare the global vaccine status and relate it with the current vaccine status (refer and cite:  doi:10.3934/publichealth.2021053)

5. Conclusion: The authors have shown the importance of variables that can influence the adherence of preventive measures and willingness to vaccinate against COVID-19.
The main bulletin messages showcases the summary of the manuscript very well.

I advocate this article for revision pending inclusion of the points as recommended by me.

We thank the reviewer for the nice comments. Moreover, we appreciate the reviewer’s suggestion and we think these references could make our research more comprehensive. The above references are indeed related to this research and we add these references in the new manuscript (in line 2, line 10, line 264, line 11, and line 13, respectively).
